# The Development and Evaluation of a Loop-Mediated Isothermal Amplification (LAMP) Method for the Detection of *Spirometra mansoni* in Dogs

**DOI:** 10.3390/vetsci13010066

**Published:** 2026-01-09

**Authors:** Xiaoruo Tan, Yuke Zeng, Shiquan Lu, Asmaa M. I. Abuzeid, Qin Meng, Zhihui Zou, Kewei Fan, Wei Liu

**Affiliations:** 1 Research Center for Parasites & Vectors, College of Veterinary Medicine, Hunan Agricultural University, Changsha 410128, China; 15178240355@stu.hunau.edu.cn (X.T.); zengyk17583177042@stu.hunau.edu.cn (Y.Z.); 123391@stu.hunau.edu.cn (S.L.); mengqin@stu.hunau.edu.cn (Q.M.); zouzhihui@stu.hunau.edu.cn (Z.Z.); 2 Fujian Provincial Key Laboratory for Prevention and Control of Animal Infectious Diseases and Biotechnology, Longyan University, Longyan 364012, China; 3 Department of Parasitology, Faculty of Veterinary Medicine, Suez Canal University, Ismailia 41522, Egypt; asmaa_ibrahim@vet.suez.edu.eg

**Keywords:** *Spirometra mansoni*, LAMP, dog feces, Hunan province

## Abstract

*Spirometra mansoni* is a zoonotic parasite that inhabits the intestines of dogs and cats. The plerocercoids (spargana) parasitize several vertebrates, including humans, resulting in a food-borne zoonosis known as sparganosis. In this study, it has been established that a LAMP assay can detect *S. mansoni* eggs in dog feces. A total of 97 fecal samples were collected from Changsha City, Hunan Province. The fecal DNA was extracted before designing primers for LAMP based on the *S. mansoni* cox1 gene. The specificity of this method was verified by PCR using LAMP outer primers or inner primers and nested PCR with *S. mansoni*-specific cox1 primers. DNA samples from five control dog worms were analyzed using the LAMP assay to evaluate the specificity. The detection rate of LAMP for *S. mansoni* eggs was 70.21% in stray dogs. PCR and nested PCR produced specific bands on agarose gel electrophoresis consistent with the expected length. When the LAMP assay was conducted using *S. mansoni*-infected samples, negative samples, and genomic DNA from control worms, only the *S. mansoni*-infected samples showed a typical ladder pattern. The samples were stained with SYBR Green I, and only the *S. mansoni*-infected samples had a fluorescent signal. In addition, compared with PCR and microscope, LAMP method can detect eggs in the shortest infection days, and its detection rate was higher than that of PCR. These results suggest that the established LAMP method have many advantages in detecting *Spirometra mansoni*.

## Simple Summary

*Spirometra mansoni* (*S. mansoni*) is a zoonotic parasite with a global distribution, infecting humans as an intermediate host. After entering the human body, spargana larvae migrate to various body parts. Adult *S. mansoni* parasitizes the small intestines of dogs and cats. There are over 50 million stray dogs and cats in China, which may accelerate the incidence of sparganosis. Epidemiological examination and clinical diagnosis of *S. mansoni* are quite limited. Common serum immunological detection methods are not suitable for popularization and low-resource applications. The dot immunogold filtration assay (DIGFA) for sparganosis detection has cross-reactions with lung fluke disease and cysticercosis. Recently, loop-mediated isothermal amplification (LAMP) has been developed. The LAMP assay was successfully employed for the specific and rapid diagnosis of several parasitic infections. However, reports about the analysis for sparganosis using the LAMP technique are limited. Here, a LAMP assay which can detect *S. mansoni* eggs within 60 min at a constant temperature has been established. The performances of LAMP assays showed clear differences between the negative control and *S. mansoni*-infected samples.

## 1. Introduction

*Spirometra mansoni* (*S. mansoni*) is the tapeworm of the genus *Spirometra*, family Diphyllobothriidae. During its life cycle, *S. mansoni* has three different hosts: final hosts (dogs and cats), first intermediate hosts (cyclops), and second intermediate hosts (frogs or tadpoles). Snakes, birds, and pigs may also act as transport hosts [1]. Humans mostly serve as intermediate hosts harboring spargana (plerocercoid larvae) [2,3]. Upon entry into human bodies, the spargana larvae migrate to various body parts, including subcutaneous tissues, muscles, brain, eyes, breasts, mouth, and lungs, causing a disease known as spargnosis. Clinical symptoms depend on the location and number of plerocercoids. The migratory subcutaneous nodules are the most common clinical feature of sparganosis. However, chills, fever, headaches, seizures, and hemiparesis may also occur [4,5]. Sparganosis is distributed globally, although primarily reported in Southeast Asia [6]. In China, sparganosis has been reported in 27 provinces, with the first case recorded in Xiamen, Fujian province, in 1882. A total of 1359 cases were detected between 1949 and 2014, mainly in eastern and southern China [5]. According to the literature reported by China National Knowledge Infrastructure (CNKI), a total of 191 cases of sparganosis were confirmed between 2015 and 2024. Based on these data, Guangdong Province had the highest number of confirmed cases, followed by Hunan Province. The sparganosis case rate in Hunan province accounted for 10.6% of the total cases in mainland China between 1959 and 2012 [7]. Nevertheless, these data may be underestimated, and the actual number of infections may be far higher because many cases may not have been recognized or reported [8]. As a result, sparganosis has already become a focus of public health in China.

China is the second-largest habitat of stray animals, with over 50 million stray dogs and cats [9]. *Spirometra mansoni* eggs were detected in the feces of dogs and cats in Guangzhou, China, with the prevalence of 27.5% and 40.5%, respectively [1]. Currently, the primary method for detecting *S. mansoni* infection is classical fecal examination. This approach has limited sensitivity and a low detection rate, challenging early diagnosis and epidemiological studies [10,11]. The commonly used serological methods consist of micro-ELISA and chemiluminescence ELISA [5]. These methods detect serum-specific antibodies against *S. mansoni*, with high sensitivity and less trauma to the donor animal. Nevertheless, ELISA typically takes a long time, especially in large-scale screening, which requires specialized, costly equipment, such as a spectrophotometer for data reading. These technical demands are not suitable for popularization and low-resource applications. Moreover, serological test cross-reactivity significantly complicates *S. mansoni* detection. For instance, 31 and 36 kDa *S. mansoni* proteins elicited specific antibody responses while showing cross-reactivity with cysticercosis [12]. ELISA employing excretory-secretory (ES) antigens had 100% sensitivity for identifying *S. mansoni* infections, but the specificity was only 96.72% when examined against other parasites [4]. The dot immunogold filtration assay (DIGFA) is a simple, rapid, and highly sensitive diagnostic method for *S. mansoni*. However, DIGFA had a high cross-reaction rate with sera of patients with paragonimiasis and cysticercosis [13], requiring additional differential diagnosis. Molecular approaches based on the polymerase chain reaction (PCR) are more sensitive and specific than parasitological and serological techniques. Recently, PCR based on the mitochondrial cox1 gene has been applied to specifically identify *S. mansoni* isolates in dogs, cats, reptiles, and amphibians [11,14]. However, these approaches are expensive due to the need for costly equipment [15]. In the LAMP reaction system, the sensitivity of the Bst enzyme to inhibitory factors in the fecal DNA template is relatively low, indicating that this enzyme exhibits more stable activity when affected by inhibitory factors. In contrast, the enzymes used in traditional PCR systems are more susceptible to interference from these inhibitory factors, which may lead to a decrease in detection efficiency. Therefore, LAMP technology, based on the conventional PCR detection method, demonstrates higher detection sensitivity [16].

Loop-mediated isothermal amplification (LAMP) is a new nucleic acid amplification method that amplifies DNA with high sensitivity, specificity, rapidity, and simplicity [17]. LAMP requires simple equipment, offers fast results, and is thus suitable for point-of-care and large-scale applications. The LAMP assay does not require a thermocycler. Interestingly, this technique can amplify DNA of low copy number to 10^9^ copies under isothermal conditions. The assay products can be visualized with the naked eye. Furthermore, this technique uses four specifically designed primers, which recognize six different fragments on the target DNA, ensuring specific amplification [18]. This technique has been successfully applied to detect pathogenic microorganisms, including parasites, such as *Opisthorchis viverrini* [19], *Toxoplasma gondii* [20], *Leishmania donovani* [21], and *Strongyloides stercoralis* [22]. In the detection of schistosomiasis, the results of the LAMP test have proved to be comparable or even superior to those of conventional diagnostic methods [23]. LAMP has been reported to be a highly sensitive and specific method for detecting parasitic infections [24]. It should be conducted more in-depth research on LAMP detection technology. Eventually, the LAMP technique may become a truly timely detection tool and an excellent option for applying molecular detection techniques to actual clinical symptoms.

The study aimed to establish a LAMP-based approach for the rapid and sensitive diagnosis of *S. mansoni* in fecal samples and estimate the detection rate of *S. mansoni* in fecal samples from dogs in Changsha.

## 2. Materials and Methods

### 2.1. Sample Collection

Fecal samples were collected from stray dogs in an animal protection organization in Changsha City, Hunan Province, using the anal swab method. Briefly, sterile cotton swabs were soaked in normal saline and inserted 3–5 cm into the anus for rotary sampling. A total of 47 valid samples were obtained and numbered from 1 to 47. Additionally, we collected fecal samples from pet dogs in several pet hospitals in Changsha City, Hunan Province, with the same anal swab sampling method, and obtained 50 valid samples. All samples were stored in amber glass vials at −20 °C.

### 2.2. DNA Extraction

A total of 200 mg of feces from each specimen was used for DNA extraction using the TIANamp stool DNA Kit (TianGen, Beijing, China) following the manufacturer’s protocol [25]. Moreover, total genomic DNA was extracted from adult control worms (*Echinococcus granulosus*, *Dipylidium caninum*, *Toxocara canis*, *Schistosoma japonicum*, and *Clonorchis sinensis*) using the TIANamp Genomic DNA Kit (TianGen, Beijing, China) according to the manufacturer’s protocol [26]. The quality and integrity of the extracted DNA samples were assessed using a NanoDrop ND-1000 spectrophotometer (Thermo Fisher Scientific, Waltham, MA, USA) and gel electrophoresis, respectively. Only DNA samples with an A260/A280 value of 1.8–2.0 were used. All DNA samples were stored at −20 °C.

### 2.3. Primer Design

Primers were designed based on the *Spirometra mansoni* mitochondrial cox1 sequence [8]. The specific primers were designed using the Primer Explorer V5 (https://primerexplorer.eiken.co.jp/e/, accessed on 10 November 2025) for LAMP and nested PCR, respectively, in the variable region of the DNA sequence. The oligonucleotide sequences of primers were shown in Table 1.

### 2.4. LAMP Assay

The reactions (25.0 μL) contained 10× ThermoPol Buffer (2.5 μL, New England Biolabs, Inc., Ipswich, MA, USA), MgSO_4_ (1.5 μL, 100 mM), dNTP (3.5 μL, 10 mM), 1.0 μL of F3/B3 primers (5 μM) and FIP/BIP primers (40 μM), Bst DNA polymerase (8 U, New England Biolabs), DNA template (1.0 μL), and distilled water (13.5 μL). A negative control reaction was prepared containing distilled water instead of the DNA template. The reaction mixtures were incubated in a heat block at 65 °C for 60 min.

### 2.5. Detection of LAMP Products

The LAMP products were loaded into a 1.5% agarose gel stained with ethidium bromide (EB), followed by electrophoresis in a Tris–acetic acid (TAE) buffer. Then, LAMP products were visualized and photographed under UV light. Moreover, we used the SYBR Green staining method to detect LAMP products. LAMP assay containing 10^−3^ SYBR Green I (TransGen Biotech, Beijing, China) was examined under UV light [27].

### 2.6. PCR Assay

PCR was conducted using LAMP outer primers (COX1-F3 and COX1-B3) and inner primers (COX1-FIP and COX1-BIP) to validate the specificity of the LAMP assay. Each reaction (50 μL) contained 25.0 μL of 2PCR SuperMix (EasyTaq/EasyPfu), 2 μL of template DNA, 1.0 μL of each primer (10 μM), and 21 μL of distilled water. The PCR cycling procedure was as follows: pre-denaturation at 94 °C for 5 min; 30 cycles of denaturation (94 °C for 30 s), annealing (55 °C for 30 s), and extension (72 °C for 1 min); final extension at 72 °C for 7 min. PCR products were analyzed using 1.5% EB-stained agarose gel electrophoresis and then visualized using UV transillumination.

### 2.7. Nested PCR Amplification

Nested PCR was conducted using *S. mansoni* cox1-specific primers (Dog-cox1-F, Dog-cox1-N, and Dog-cox1-W), shown in Table 1. The PCR was performed in an automatic thermocycler using a total reaction volume of 50 μL, containing 25.0 μL of 2PCR SuperMix (EasyTaq/EasyPfu), 0.5 μg of template DNA, 1.0 μL of each primer (10 μM), and distilled water up to 50 μL. The primary PCR parameters were as follows: 94 °C for 5 min; 30–35 cycles at 94 °C for 30 s, 55 °C for 30 s, and 72 °C for 1 min; 72 °C for 7 min. PCR products were analyzed using 1.5% EB-stained agarose gel electrophoresis and then visualized using UV transillumination.

### 2.8. Evaluation of LAMP Specificity

To evaluate the specificity of the LAMP assay, common dog worms (*Echinococcus granulosus*, *Dipylidium caninum*, *Toxocara canis*, *Schistosoma japonicum*, and *Clonorchis sinensis*) were selected as controls. Total genomic DNA samples from adult control worms were used as a template for the LAMP assay.

### 2.9. Detection Result Analysis

The dog fecal samples (47 stray dogs and 50 pet dogs) were analyzed using the DNA extraction method and LAMP assay amplification. PCR products were examined using 1.5% EB-stained agarose gel electrophoresis and UV transillumination. The 95% confidence interval was calculated using SPSS v.24.0 (https://www.ibm.com/products/spss-statistics, accessed on 10 November 2025).

## 3. Results

### 3.1. Results of Conventional PCR and Nested PCR

**Figure 1:**
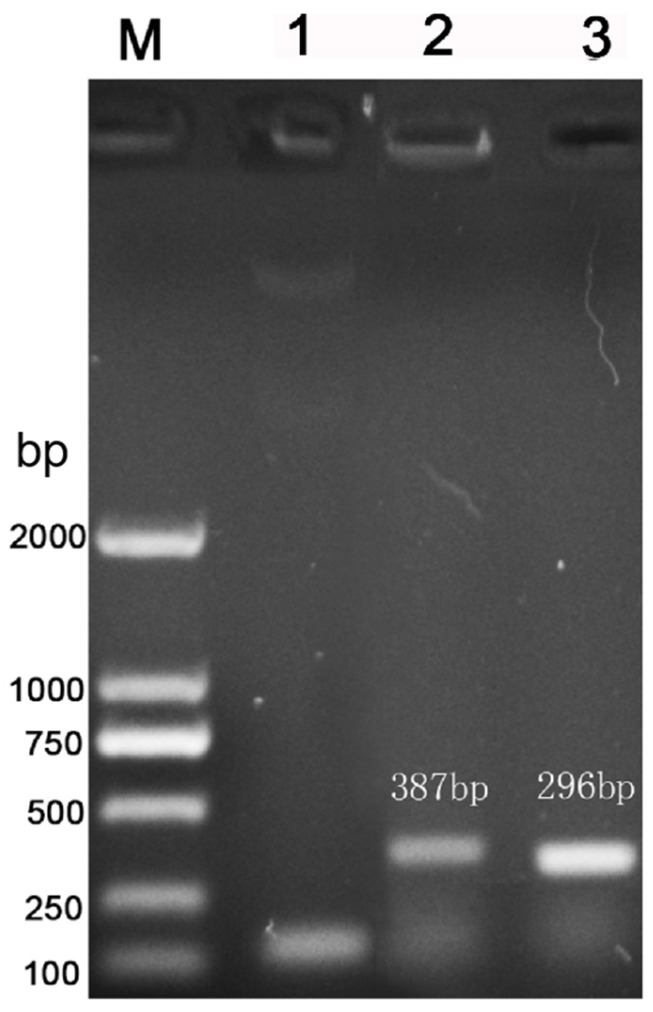
The outer and inner primers of extracted DNA. Lane 1, nested PCR amplification of *S. mansoni* cox1 gene; Lane 2, PCR product obtained using LAMP outer primers; Lane 3, PCR product obtained using LAMP inner primers.

The *Spirometra mansoni*-specific cox1 fragment was amplified by nested PCR, showing a specific band on agarose gel electrophoresis in lane 1 (Figure 1). PCR amplification of a *S. mansoni*-infected fecal sample using LAMP outer primers and inner primers produced specific bands consistent with the expected lengths of 387 bp and 296 bp, respectively (Figure 1).

### 3.2. LAMP Specificity and Sensitivity Assay

**Figure 2:**
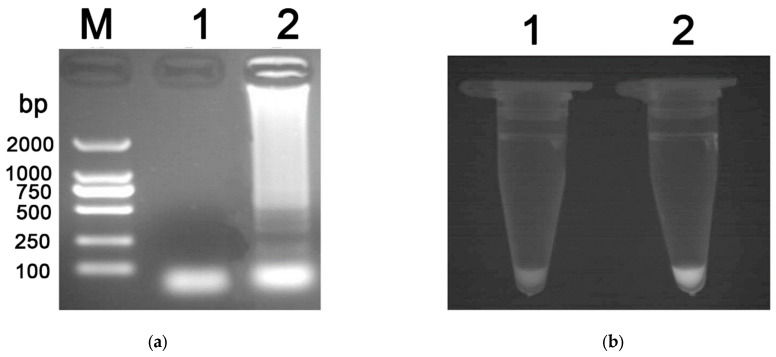
Specificity of the LAMP assay. (**a**) The electrophoretic results of LAMP products. Lane 1, the negative control; Lane 2, the typical ladder pattern of *S. mansoni*-infected sample; (**b**) The SYBR Green I staining of LAMP products. Lane 1, the negative control; Lane 2, *S. mansoni*-infected sample.

DNA was extracted from dog fecal samples from Hunan Province to assess the specificity of the LAMP assay. A negative control containing distilled water was also set. Results of gel electrophoresis showed that only the *S. mansoni*-infected LAMP products formed a typical ladder pattern (Figure 2a). However, the negative control showed no ladder under the UV transillumination. When the two samples (*S. mansoni*-infected and negative) were stained with SYBR Green I, fluorescent signals were observed in *S. mansoni*-infected reactions without opening the tubes (Figure 2b).

**Figure 3:**
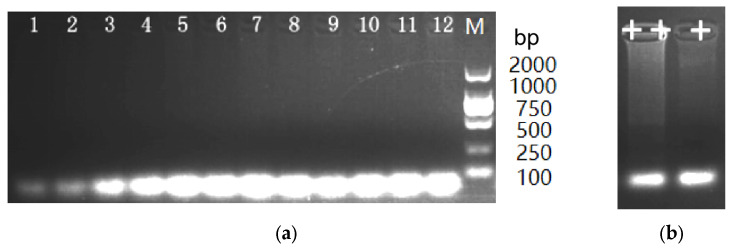
LAMP results of *S. mansoni*-infected sample and control worms. (**a**) Gel electrophoresis results of LAMP products from other parasite samples: Lanes 1–3, *Echinococcus granulosus*; Lanes 4–6, *Dipylidium caninum*; Lanes 7–8, *Toxocara canis*; Lanes 9–10, *Schistosoma japonicum*; Lanes 11–12, *Clonorchis sinensis*; (**b**) Gel electrophoresis results of LAMP products from *S. mansoni*-infected samples.

Based on the aforementioned reaction conditions, the LAMP assay was performed using DNA samples from *Echinococcus granulosus*, *Dipylidium caninum*, *Toxocara canis*, *Schistosoma japonicum*, and *Clonorchis sinensis*. The results were shown in Figure 3a. None of these samples showed a ladder pattern during amplification. *S. mansoni*-infected LAMP products showed a typical ladder pattern (Figure 3b). These results suggested that the LAMP detection method had high specificity in detecting *Spirometra mansoni*.

The genomic DNA of *S. mansoni* was quantified to 10 ng/µL and diluted 10-fold. Using different concentrations of genomic DNA as templates, LAMP and PCR amplifications were performed, respectively. The results showed that both LAMP and PCR methods could detect 10^−3^ ng, with the same sensitivity. However, after detecting the fecal DNA of infected dogs at different infection days, it was found that the LAMP method could detect *S. mansoni*-infected results on the 8th day after infection, while PCR could detect infected results on the 10th day after infection. The microscope could only detect the eggs on the 12 day after infection. LAMP method and PCR technology were used to detect the collected dog feces samples. The results showed that the detection rate of LAMP was 70.21% and the detection rate of PCR was 68.09% in 47 stray dog feces samples. However, the detection rate of pet dog feces samples by two methods is 0. Thus, it can be seen that the LAMP detection method established in this study has higher sensitivity in detecting the fecal DNA of infected dogs.

### 3.3. Detection Result Investigation

Table 2 showed the applicability of the LAMP assay in detecting *S. mansoni* in dogs using fecal DNA templates (Table 2). Out of 47 fecal samples from stray dogs, the proportion of *S. mansoni*-infected samples (detection rate) was 70.21%. The 95% confidence interval of the detection rate was 56.93% to 83.49%. This result suggests that there was a 95% probability that the infected rate would fall within the range of 56.93% to 83.49%.

According to the results of LAMP detection and PCR detection, the contingency table was constructed (Table 3). After calculation, the observed consistency (Po) was about 0.894, the expected consistency (Pe) was about 0.573, and finally the Kappa coefficient (K) was about 0.751, indicating that the two detection methods had good consistency.

However, unexpectedly, LAMP assay of fecal samples collected from several pet hospitals in Changsha City, Hunan Province, showed that there were no *S. mansoni*-infected samples in pet dogs.

## 4. Discussion

Here, it has been established a LAMP assay for the sensitive detection of *S. mansoni* eggs, helping early diagnosis and control of this parasite. The LAMP detection method usually completes the reaction at a constant temperature (typically 60–65 °C), and the required time is within 1 h. In contrast, PCR requires the pre-denaturation, denaturation, annealing, extension and the number of cycles in the reaction program to complete the reaction. Although the LAMP method has the same sensitivity as PCR when conducting concentration dilution tests on genomic DNA, it can detect eggs even when the infection duration is the shortest. Furthermore, the experimental results of the LAMP detection also indicate that there are significant differences between the *S. mansoni*-infected samples, the negative samples, and the other parasite samples. The established LAMP detection technology is more convenient, saves time, and has the significance of early diagnosis. It also has higher specificity and sensitivity.

Sparganosis has been reported to have a higher prevalence in several Asian countries, including South Korea, Japan, Thailand, and China [28,29,30]. Carnivores, such as dogs and cats, serve as the final hosts of *S. mansoni*, which carry the adult worms in their small intestines [31]. In earlier reports in China, the prevalence of *S. manoni* eggs in the feces of dogs and cats was 1.48% in Jiangsu Province [32], 0–47.16 (27.5%) in dogs and 0–64.44 (40.5%) in cats in Guangdong Province [1], 0–5% in dogs and 0–15% in cats in Huainan, Anhui Province, and 19.4% in dogs and 30.3% in cats in Luohe, Henan Province [33]. However, infection status investigations of *S. mansoni* in Hunan Province are lacking. In this study, the *S. mansoni*-infected detection rate of the LAMP assay in fecal samples from stray dogs in an animal shelter in Changsha, Hunan Province, exceeded 70%. Although a moderately high prevalence (47.16%) was recorded in dogs in Yunfu, Guangdong [1], previous reports in China [32,33] based on traditional fecal examinations in dogs recorded *S. mansoni* prevalence lower than that recorded in our study using the LAMP technique. This difference may be related to the difference in geographical distribution and the higher sensitivity of the LAMP assay compared to traditional fecal tests. Several studies suggested that LAMP could detect extremely low quantities of parasite DNA (as little as a single egg or trophozoite), exceeding the detection limits of microscopy and even conventional PCR [34,35,36]. LAMP can be useful in detecting low-burden or post-treatment infections that are usually missed by fecal microscopy or egg counts [36,37]. There are a large number of stray animals in China, exceeding 50 million stray dogs and cats [9], which might increase the risk of sparganosis. Surprisingly, in this study, the *S. mansoni*-infected detection rate of fecal samples from pet dogs provided by several pet hospitals in Changsha, Hunan Province, was 0%. It was speculated that the scientific pet-keeping awareness of most pet owners has significantly improved nowadays. They usually give their dogs worm treatments on time, thereby effectively reducing the risk of contracting the *S. mansoni*.

The clinical diagnosis of sparganosis is often misdiagnosed due to the absence of specific clinical signs and predilection sites for the larvae. Recently, several methods have been developed for detecting *S. mansoni* in diseased animals. The ELISA with excretory–secretory (ES) antigens of spargana had a high sensitivity for sparganosis serodiagnosis [38]. However, the main limitations of this assay are the cross-reactions with sera from patients with other parasitic diseases, such as clonorchiasis, paragonimiasis, schistosomiasis, cysticercosis, and echinococcosis [38]. Additionally, ELISA is also problematic in terms of time and cost. Virtually, DIGFA is considered a cost-effective and easy method for field screening. Using DIGFA, Chen et al. [39] conducted a rapid serodiagnosis of cystic hydatid disease caused by the larval stage of *Echinococcus granulosus*. Eosinophilic meningitis caused by *Angiostrongylus cantonensis* was also diagnosed by DIGFA [40]. Unfortunately, DIGFA has the disadvantage of false-positive or cross-reactions of *Spirometra mansoni* with paragonimiasis and cysticercosis [13]. As a result, there are some limitations in both ELISA and DIGFA for determining sparganosis.

It has been suggested that, in contrast to PCR, LAMP is not adversely affected by irrelevant target DNA. Moreover, LAMP can amplify a large quantity of the target DNA under isothermal conditions in 60 min, thus being faster than traditional PCR [17]. Recently, it has been increasingly reported that the LAMP technique was successfully applied to the rapid and specific detection of protozoa and helminth infections, such as *Toxoplasma gondii* [20], *Leishmania donovani* [21], *Wuchereria bancrofti* [41,42], *Necator americanus*, *Strongyloides stercoralis* [22], *Opisthorchis viverrini* [19], *Clonorchis sinensis* [35], and *Fasciola hepatica* [37]. However, published reports about the analysis for sparganosis using the LAMP technique are deficient. In the present study, the LAMP method was successfully established to detect *Spirometra mansoni* in dogs. To determine the specificity of the *S. mansoni* LAMP assay, nested PCR reactions were conducted for amplification of the *S. mansoni*-specific cox1 gene. Moreover, PCR was performed using outer and inner primers to assess the LAMP-infected samples, ensuring that the LAMP assay amplified the target DNA and thereby confirming high specificity to *S. mansoni*. The amplicon sizes of conventional PCR and nested PCR were consistent with the expected size. Moreover, the established LAMP assay was conducted using *S. mansoni*-infected samples and negative controls (distilled water) and analyzed using EB-stained gel electrophoresis and SYBR green staining. We also utilized control DNA samples from common helminths that discharge their eggs and/or gravid segments in dog feces, including *Echinococcus granulosus* [43], *Dipylidium caninum* [44], *Toxocara canis* [45], *Schistosoma japonicum* [46], and *Clonorchis sinensis* [47]. None of the control samples (negative control or control worms) showed the LAMP characteristic ladder pattern on gel electrophoresis. Although these results showed that LAMP method had many advantages in detecting *Spirometra mansoni*, this study mainly used this technology to detect *Spirometra mansoni* and other susceptible parasites in dogs and common parasites in daily life. Therefore, it is still necessary to further study and use more kinds of samples in different geographical areas to verify the effectiveness of this method.

## 5. Conclusions

This study developed and applied a LAMP assay to detect *S. mansoni* eggs in dog fecal samples, with a detection rate of 70.21%. This assay was specific to *S. mansoni* compared to five control dog worms. Compared with PCR method and microscope, LAMP method could detect more quickly and simply. This study may promote rapid clinical diagnosis and epidemiological investigation of *Spirometra* species, contributing to the prevention and control of sparganosis.

## Figures and Tables

**Table 1:** The sequences of primers of LAMP and nested PCR.

Name	Sequences	Test
COX1-F3	TTTACTGTGGGGTTGGAC	LAMP
COX1-B3	CAAGCAGAAAGAATTATACCAGT	LAMP
COX1-FIP(F1C-F2)	ACCTTTATACCCGTGGGAACCGAATTCAGACTGCTGTTTTCTTTAGTTCT	LAMP
COX1-BIP(B1c-B2)	AATAGTCGTGTTTCGTTGCGTGAATTCCACCCATAGTAAACAACACAAT	LAMP
Dog-cox1-F	TTTGGGCATCCTGAGGTTTATG	Nested PCR
Dog-cox1-N	TCACGCAACGAAACACGACT	Nested PCR
Dog-cox1-W	TTTATCCAACACACAAGCAG	Nested PCR

**Table 2:** Examination of LAMP products of fecal samples from stray dogs in Changsha City (*n* = 47).

Sample ID	1	2	3	4	5	6	7	8	9	10	11	12	13	14	15	16
Results	−	−	+	+	−	+	+	−	−	+	+	+	−	+	−	−
Sample ID	17	18	19	20	21	22	23	24	25	26	27	28	29	30	31	32
Results	+	+	+	+	+	+	+	−	+	+	+	+	+	+	+	+
Sample ID	33	34	35	36	37	38	39	40	41	42	43	44	45	46	47	
Results	−	+	+	+	+	+	+	−	−	+	+	+	−	−	+	

+, *S. mansoni*-infected reaction; −, negative reaction.

**Table 3:** Construction of contingency table based on LAMP detection and PCR detection.

	PCR Positive	PCR Negative	Total
LAMP positive	30	3	33
LAMP negative	2	12	14
Total	32	15	47

## Data Availability

The original contributions presented in this study are included in the article. Further inquiries can be directed to the corresponding authors.
